# Intelligent Light Control System Based on Zigbee

**DOI:** 10.1155/2022/3814510

**Published:** 2022-03-19

**Authors:** Xinxin Wang, Lijun Wang

**Affiliations:** School of Mechanical Engineering, North China University of Water Resources and Electric Power, Zhengzhou, China

## Abstract

This system uses Freescale i.MX283 chip as the main control core of the control panel and LPC824 as the main control core of the light control point. ZigBee communication local area network is built. The ZigBee network is used to search all the nodes and display them on the control interface stably. The controller can selectively control the node and communicate with ZigBee. It supports simple and convenient human-computer interaction interface. The control panel can control RGB lamps of lamp control nodes as follows: control the light on and off, adjust the brightness of the light, toggle the color of the light, control the gradient of the light, control the flashing of RGB lamp, control timing switch of RGB lamp, realize the scene effect of the light, etc. After testing, the system is stable and applied to many lighting control occasions.

## 1. Introduction

With the rapid development of science and technology, the development trend of wireless communication combined with intelligent control systems is more and more obvious, such as smart homes and the Internet of things. They all take the system as a carrier and rely on a way of communication to control the state and exchange data. The control side controls the state (foreground effect), and the node changes its state (background effect). The system mainly processes and calculates various complex data and realizes the algorithm. Communication is mainly used for data transmission and the exchange of feedback. In this way, it can not only realize highly complex algorithm functions but also support a human-computer interaction interface which is convenient for users. The intelligent lighting control system based on ZigBee communication integrates computer technology, mechanical control theory, sensor technology, system engineering, and artificial intelligence technology. It has a wide range of applications and is the development trend of Internet of things communication in the future [1].

## 2. Overall Design Scheme

This design selects i.MX283 as the control interface; its core is an ARM9 processor. The processing speed is fast enough because it has enough RAM space; it can also run common operating systems such as UCOS, LINUX, and AWorks. It has its own ZigBee minimum system module supporting the ZigBee protocol. The LPC824 of Freescale is selected as the control node, and the processor is arm CortexM0+ which is a low-power controller with a processing speed of about 30 m. For the light control node, this speed is large enough, and it has its own ZigBee minimum system module that supports ZigBee protocol and can realize various state controls of RGB [2].

The software design is mainly divided into control panel and light control node. The control panel provides a simple human-computer interaction control interface for users who can click the interface control icon to send out the corresponding control signal according to the needs of their own functions. Then, the data layer will send the control signal to the light control node for execution through ZigBee protocol communication. The light control node continuously queries and receives the control signal sent by the control panel and then analyzes the command and executes the corresponding control command to achieve the corresponding functions.

The control panel mainly involves five thread tasks [3]:

1. GUI tasks: used to create and display function icons. When the user clicks the function icon, the callback function will be called to complete the corresponding function. For example, select and switch the target control node, send the control status command, receive the temperature and humidity data, mailbox data, and real-time display, receive the surrounding node information and display node-related information, and modify the display name of the node to facilitate memory.
2. Temperature and humidity acquisition task of DHT11: mainly collect the current temperature and humidity, and send the data to the GUI interface through the temperature and humidity data mailbox.
3. Receive control signal task of light control node: receive the control information data from the GUI task layer in real time, and then, the task is sent by ZigBee.
4. ZigBee search task: search the surrounding node information, and send all node information to GUI.
5. ZigBee sends control status task: sends control information data to the lamp control node.
6. Light control node task: the light control node is equivalent to a state machine. ZigBee protocol is used to receive the control information from the control interface in real time and then analyze and execute the control information.

## 3. Software Design

The software development of intelligent lighting control system is based on eclipse software as the development environment, C language as the main programming language, and Freescale chip ARM9 and cortex M0+ as the core processor. The software module needs to use the task allocation, task scheduling, communication mechanism between tasks, GUI, GPIO port control, UART module, PWM module, and ZigBee communication protocol of real-time system AWorks.

### 3.1. ZigBee Networking and Communication Process

The coordinator can create a unique WLAN PANID. Routers and terminals can join a wireless LAN PANID, that is, network access. The PANID created by the coordinator and the PANID added by the router and terminal are preset in the program and are not automatically allocated by the module. Therefore, in the process of network creation and network addition, it is necessary to specify which LAN to create or need to join, so as to ensure the effectiveness of data transmission.

The coordinator module establishes the wireless local area network. This PANID is unique. When selecting the channel, select the channel without a local area network nearby (there are 16 channels in 2.4 G bandwidth to choose from 11 to 26) which can prevent competition and increase the stability of the signal [4].

When the coordinator creates a LAN and a module (router or terminal) joins the LAN, the node can communicate with the router. Terminals cannot communicate directly with each other and need to pass through routers to act as an intermediary. Sending data must comply with ZigBee protocol and can be developed using ZigBee protocol stack provided by ZigBee manufacturer. The protocol stack is like a development library written according to the provisions of the ZigBee protocol. Developers only need to develop through the API functions provided by the library functions so as to realize the sending and receiving of wireless data [5].

### 3.2. Interface Layer Design of the Control Panel

#### 3.2.1. GUI Control Interface

emWin and ucGUI are the same; GUI graphics library is like a function encapsulation library which provides developers with various interfaces for drawing API. Understand API functions and precautions to carry out preliminary development, and emWin interface application structure is clear; each page is composed of three parts: (1) control structure array, (2) callback function, and (3) page creation function. Then, there is the window and control management which are mainly composed of (1) control handle, (2) control ID, and (3) message [6, 7].

*(1) Control Array*. View all the controls used in the current interface. The main function is to create and initialize the information of related controls such as the abscissa and ordinate of the control displayed in the current interface, the size, and name of the control.

*(2) Callback Function*.

  static void _dialog(massge *∗*p_msg) {
  switch (p_msg- > id) {
  case init://Reset control properties (size, color, etc.)
  case parent://Operations trigger message processing such as clicking the//button, clicking the edit box
  case timer://Timer trigger operation, timer will execute the program here
  case paint://Window redraw message, display text in the window or draw lines,//rectangles, circles
  }
  }
  Page creation function:
  void task_ main(void)
  {
  gui_ Init();//initialization interface
  create_ Win();//create interface
  while (1) {delay (20); }//call GUI_ Delay function delay 20 ms
  }

Each page has its own callback function which contains all the functions that the control needs to implement. Any operation of the interface will trigger and call this callback function and then process it in the program.

#### 3.2.2. GUI Interface Design

The intelligent light control system based on ZigBee mainly includes the display and selection of node, the switch of node light, the color of light, the brightness of light, the flicker of light, the scene effect of light, the timing of light, and the gradient of light. The control selection mainly includes button control, checkbox control, slider control, and text control [8, 9].

*(1) Creation and Start of Interface*.

  void gui_task(void *∗*p_arg)/*∗* GUI task function entry *∗*/
  {
  WM_HWIN Win;
  GUI_Init();/*∗* Initialization interface *∗*/
  Win = CreateSmsrt_Control(WM_HBKWIN);/*∗* Create interface *∗*/
  while(1) {
  GUI_Delay(20);/*∗* Waiting for task scheduling *∗*/
  }
  }

Button control is used for the selection of light color. When user presses button control, GUI task will call callback function to create a color selection interface.

The program framework of button control is as follows:

  static void dialog(massge *∗* p_msg) {/*∗* GUI task callback function *∗*/
  switch (p_msg - > msg_id) {/*∗* Type judgment of callback *∗*/
  case notify_parent:/*∗* The callback type is operation trigger message *∗*/
  id = get_id(p_msg - > win_src);/*∗* Get interface control type*∗*/
  ncode = p_msg - > data.v;/*∗* Operational data *∗*/
  switch(id) {/*∗* Control type selection *∗*/
  case botton_0:/*∗* botton_0 Control type *∗*/
  switch(ncode) {
  case click:
  break;
  case reaessed;/*∗* Click operation *∗*/
  create_colo_win();/*∗* Create a color selection interface *∗*/
  break;
  }
  break;
  }}}

*(2) Checkbox Control*. Switch for light, scene effect, timing function, and gradient function. It is equivalent to a check box. Open a function (such as scene effect function); click the scene effect option box to check it. Then, the GUI task will call the callback function to execute the selected condition options of the checkbox control, and the LED status data mailbox will be sent out to control the lighting effect of the light control node [10, 11].

The program framework of the checkbox control is as follows:

  static void dialog(massge *∗* p_msg) {/*∗* GUI task callback function *∗*/
  switch (p_msg - > msg_id) {/*∗* Type judgment of callback *∗*/
  case notify_parent:/*∗* The callback type is operation trigger message *∗*/
  id = get_id(p_msg- > win_src);/*∗* Get interface control type *∗*/
  ncode = p_msg - > data.v;/*∗* Operational data *∗*/
  switch(Id) {/*∗* Control type selection *∗*/
  case check_1:/*∗* check_ 1 control type *∗*/
  switch (ncode) {
  case click:
  break;
  case reaessed:{/*∗* With ID_ CHECKBOX_ 1 click operation *∗*/
  send_mailbox(mail_type,&data, wait_time);/*∗* Send various data messages *∗*/
  break;
  }
  break;
  }} }

*(3) Slider Control*. Used for brightness adjustment of light and flicker frequency adjustment of light. It is like people seeing progress bars, but it has its own length range and size. It is like a ruler; drag it randomly, and the current dimension value will be represented at the last stop. Then, the size returned by the ruler can be used to adjust the intensity and flicker frequency of the RGB lamp which is proportional to the length of the size [12, 13].

The framework of the glider control is as follows:

  static void dialog(massge *∗* p_msg) {/*∗* GUI task callback function *∗*/
  switch (p_msg - > msg_id) {/*∗* Type judgment of callback *∗*/
  case notify_parent:/*∗* The callback type is operation trigger message *∗*/
  id = get_id(p_msg- > win_src);/*∗* Get interface control type *∗*/
  ncode = p_msg - > data.v;/*∗* Operational data *∗*/
  switch(id) {/*∗* Control type selection *∗*/
  case slider_1:/*∗* SLIDER _ 1 control type *∗*/
  switch (ncode) {
  case clicked:
  break;
  case released:/*∗* SLIDER _ 1 click operation *∗*/
  hItem = get_dialog_item(p_msg- > win, slider_1); /*∗* Get handle *∗*/
  slider_set_width (hitem,15);/*∗* Sets the size of the slider *∗*/
  slider_set_range(hitem,0,9);/*∗* Set the scale range of slider *∗*/
  val = slider_get_value(hitem);/*∗* Gets the current size value *∗*/
  send_mailbox(mail_type,&data, wait_time);/*∗* Send data message *∗*/
  break;
  }
  break;
  }}}

*(4) Timer Mechanism of GUI*. GUI handles corresponding events through the callback function. When time is up, it will execute an option callback function of the timer, and the timer will be set once. When the timer is used again, the timer count must be enabled again. This design mainly has two timers: one is responsible for the timing, receiving temperature and humidity data information every 2 seconds, and displaying it on the interface. The other is responsible for receiving the number of surrounding nodes every 3 seconds, dynamically increasing and deleting the number of nearby nodes, and displaying them on the interface [14].

Timer program framework is as follows:

  static void dialog(massge *∗* p_msg) {/*∗* GUI task callback function *∗*/
  switch (p_msg - > msg_id) {/ *∗* Type judgment of callback *∗*/
  case timer:/*∗* The callback type is timer *∗*/
  if (get_timer_id(p_msg - > data.v) = = 0) {/*∗* TIMER0 *∗*/
  receive_mailbox(mail_type, p_data,wait_time);/*∗* Receive temperature and humidity data mailbox *∗*/
  restart_timer(p_msg - > data.v, 2000);/*∗* Restart the timing for 2 seconds*∗*/
  }
  if (get_timer_id(p_msg - > data.v) = = 1) {/*∗* TIMER 1 *∗*/
  receive_mailbox(mail_type, p_data,wait_time);/*∗* Receive data mailbox of surrounding nodes *∗*/
  restart_timer(p_msg - > data.v, 3000);/*∗* Restart the timing for 3 seconds*∗*/
  } }}

### 3.3. Design of Data Layer of the Control Panel

**Figure 1:**
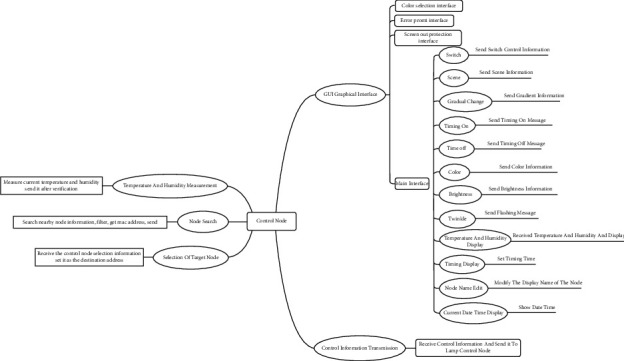
General structure of the control panel.

This system needs frequent data transmission and data transmission between tasks. So the data processing layer is designed. All data will be collected, sent, or accepted directly or indirectly through the data layer. The main data involved are temperature and humidity data, status command data of light control node, number of surrounding nodes, control information data sent by ZigBee, search node queue data, current node information list, and modified name node information list. The main tasks involved are as follows: ZigBee searches the information of nearby nodes, sends the control data to the light control node, receives the light control node data from the interface layer, and collects the temperature and humidity in real time and sends it to the interface layer and interface touch screen [15, 16]. The overall structure of the control panel is shown in Figure 1.

#### 3.3.1. Realization of Touch Screen Tasks

**Figure 2:**
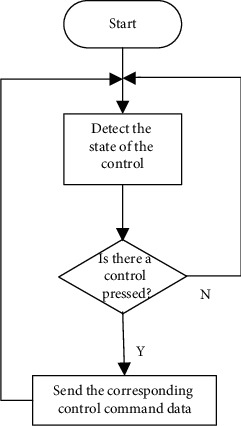
Flow chart of touch screen detection.

It mainly completes the initialization and calibration of LCD and monitors whether there are touch screen operations in real time. When there are touch screen operations, it will call the interface callback function to perform the corresponding functions. Its task level of 2 is the highest so as to successfully monitor all touch screen operations [17]. The flow chart of touch screen detection is shown in Figure 2.

#### 3.3.2. Realization of Temperature and Humidity Real-Time Acquisition

According to the protocol specification of DHT11, read the measured temperature and humidity and then send the data to the interface layer display by e-mail through intertask communication. Mission priority is 7. The driving steps of the DHT11 temperature and humidity sensor are as follows:

1. After the DHT11 module is powered on, wait for a second before sending the instruction to the module and set the data pin of DHT11 to the input state to detect the external signal.
2. The controller sends out a start signal (low level) and then waits for the temperature and humidity module to respond to the controller [18].
3. After the temperature and humidity module generates the response signal, the high level of 80 microseconds is output to tell the controller to prepare the data to receive.
4. After the preparatory work is completed, DHT11 starts to send data to the controller; the controller only needs to receive it. The data output by DHT11 has 40 bits including 16 bits of temperature data, 16 bits of humidity data, and 8 bits of calibration data.

*(1) Data Format*. Because DHT11 adopts single bus communication, it can only transmit data in one direction at the same time. It transmits bit by bit (generally used to transmit digital data). Because data are transmitted through high and low levels, it is necessary to specify the form of data. The format of data “0” is 50 ms low level +  28 ms high level and the format of data “1” is 50 ms low level +  70 ms high level.

*(2) Controller Parsing Data*. The data received by the controller is continuous level, so developers need to convert the level into effective data. According to the data format, there are many kinds of parsing methods. The method used in this design is as follows: first, wait for the low level to pass, that is, wait for the data line to pull high, then delay for 60 us, because 60 us is greater than 28 us and less than 70 us, and then detect whether the data line is high at this time. If it is high, the data is determined as 1; otherwise, it is 0 [19, 20].

**Figure 3:**
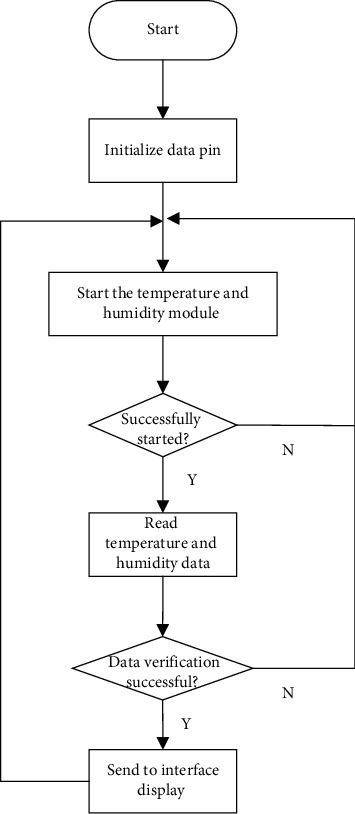
Flow chart of temperature and humidity measurement and display.

*(3) Precautions*. There will be a lot of delay waiting in the process of starting the temperature and humidity sensor and reading the data. Active delay in the operating system causes the operating system to perform other tasks with high priority. If the temperature and humidity measurement task is delayed, but the operation system does not return (because there are high priority tasks to be processed), the temperature and humidity data read will be incorrect, and the verification will not be successful in the final data verification. There are two solutions. Method 1: when entering the task of measuring temperature and humidity, the priority of this task is set to the highest; then, it will be lowered back to the original priority after measurement is completed. Method 2: when entering the task of temperature and humidity measurement, pause the task switching, then turn on the task switch after the measurement is completed. Of course, they will have a certain impact on the whole system. The most obvious consequence is that users will obviously feel that the response of the system is not timely. Finally, combined with their advantages and disadvantages, this system did not use these two methods, because the indoor temperature will not change very fast, and it only needs another 30 seconds to have a successful test to meet the actual needs [21]. The flow chart of temperature and humidity measurement and display is shown in Figure 3.

#### 3.3.3. Realization of ZigBee Search Nearby Node Information Task

**Figure 4:**
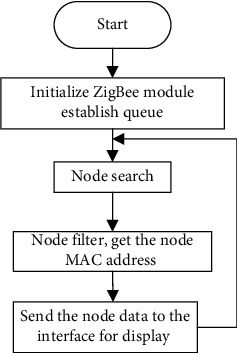
Flow of searching for nearby node information.

When users want to control a node, they first need to retrieve the existence of the node nearby. Before ZigBee search nodes, some parameters of ZigBee need to be set such as channel of the network, PANid of the network, and the source address of target address. The design sets 2.4 G of network channel, 11 network channels, 1010 network IDs, and 2020 source addresses, in which the target address can be dynamically switched according to the control target. After configuring the basic information, search constantly the number of nearby nodes and their address information in the task and then send the received number of nodes and node address information to the interface layer for processing. It is worth noting that ZigBee is also a peripheral resource. When a task is using ZigBee to send messages, the ZigBee module needs to be protected. At this time, another task can no longer search the information of nearby nodes through the ZigBee module. So it is generally to add a lock to lock to the ZigBee module and prevent other tasks from accessing it to prevent competition. The flow of search for nearby node information is shown in Figure 4.

#### 3.3.4. Realization of Node Information Display Task

**Figure 5:**
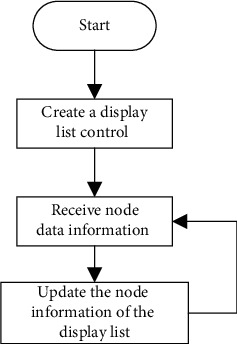
Flow of showing current node information.

After searching the nearby nodes, it needs to be displayed on the control interface to control them. According to the row comparison between the node currently searched and the node last displayed, if there is a node in the last display list but not in the current search, it will be deleted from the display list. If there is no node in the last display list but there is a node in the current search, it will be added to the display list for display [22]. It is displayed in a list box control of emWin. The flow of showing current node information is shown in Figure 5.

#### 3.3.5. Receive Interface Layer Sends Out the Control Status Task of Light Control Node

**Figure 6:**
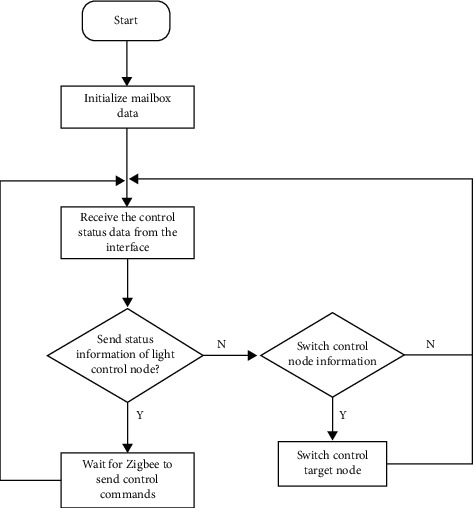
Flow of receive control command and send to node.

The purpose of this task is to receive the light control status information sent by the interface layer in real time. Concentrate the control status here and prepare to send it to the light control node, for example, switch, node selection, light brightness, light intensity, and so on. The priority is high, and the task level is 4. The receiving of light control status information is mainly in the form of a mailbox. First, create a mailbox data type that controls the status information type and set the number of mail. Finally, initialize and allocate memory space. It should be noted whether the sender has blocked sending, that is, whether he has been waiting for the successful sending and has been received by the receiver, or just send without waiting. The receiver is similar. This design uses sender blocking, receiver not blocking, and receiver real-time view receiving mailbox, if there is a mailbox to receive, not ready to receive the next time. The flow of receive control command and send to node is shown in Figure 6.

#### 3.3.6. Modifying Node Name Task

**Figure 7:**
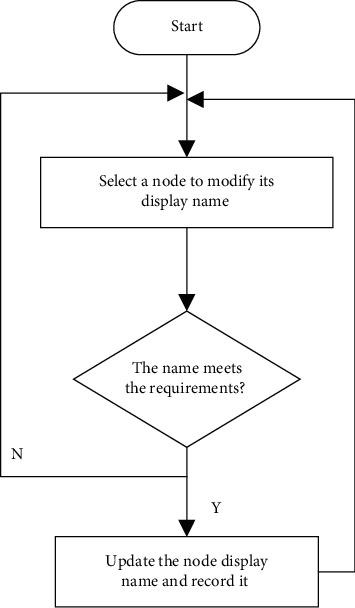
Flow of renaming node names.

Because the node is displayed in the control panel in the form of the network address of the node, and the network address is in the form of a 16-byte number, the node that may be seen on the interface is displayed in the form of a hexadecimal byte similar to 02030507, which will be very inconvenient for customers to use. Do not recognize where this node is or what special function it has from its name. In order to facilitate the use, set the name of the node that users can understand such as the living room and bedroom so that you easily identify and distinguish nodes. The flow of renaming node names is shown in Figure 7.

### 3.4. Data Layer Design of Light Control Node

#### 3.4.1. Function Overview

**Figure 8:**
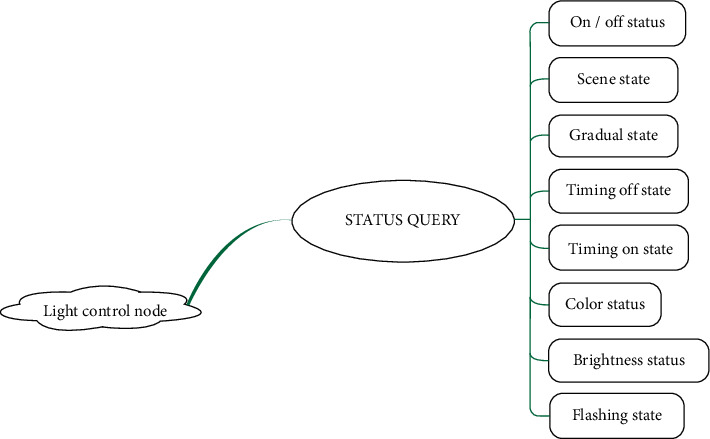
Light control node structure.

The light control node is the controlled terminal equipment which is placed in any room or outdoor and controlled through the control panel to achieve the corresponding functions. The main functions are light on and off, light gradient, adjustable frequency flicker of light, timing switch, scene effect, brightness adjustment of light, and the color setting of light. The light control node structure is shown in Figure 8.

#### 3.4.2. Function Realization of Light Control Node

**Figure 9:**
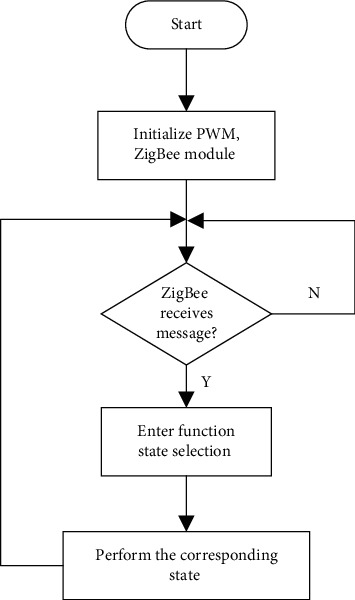
Light control node state machine.

The software design of the light control node is like an infinite cycle state machine. Although it does not have the support of the operating system, it can continuously use the ZigBee module to receive the control signals from the control panel. Then, the control signal is parsed into the corresponding control command, entering the status query. Then execute the corresponding state function, that is, each function module that the light control node can achieve. These functions are to be debugged separately and sorted and packaged after debugging [23]. State machine implementation block diagram of light control node is shown in Figure 9.

## 4. System Debugging and Optimization

### 4.1. Communication Debugging between Multitasks

When programming in a system without an operating system, two different functions also need to be exchanged generally through the way of global variables to exchange data. Generally, data exchange is carried out through global variables. When ZigBee searches the current number of surrounding nodes and information, it needs to send the information to the interface task for display. Before sending, the data will be printed out by serial port printing. After receiving the data, the interface will print out the data which is always 0. The problem is that the memory here is cleared after sending which causes the received data to be always 0. Finally, the memory space will be cleared after receiving the data.

In the process of multitasking, when different tasks use the same hardware device at the same time, it needs to be protected artificially. The most common method is to lock the module or memory and unlock it after accessing it. When the ZigBee module searches for nearby nodes, due to the scheduling of tasks, it is possible to send ZigBee information. At this time, there will be two tasks accessing the ZigBee module at the same time which will seriously lead to the death of the task and slightly lead to errors in the search or the sending of information which can not achieve the expected effect of the program. This module needs to be protected. When task A accesses this module, it will lock this module. At this time, if task A is scheduled to execute another task B, it will wait until task A's access ends when it also needs to access this module. After task A is unlocked, Task B can have the opportunity to access this module which avoids the problem of multiple tasks accessing the same module at the same time. During debugging, it is found that ZigBee sending task is not executed.

In the process of node display and name change debugging, two identical nodes will be displayed. Sometimes, when the node name is changed, it still displays the previous node name. The solution is to create two linked lists. Linked list A records the node information currently displayed; linked list B records which node names have been changed. First, the node searched is compared with list B. The name of the node is changed. The node searched is compared with list B and updated displayed. When the node name is modified, the information and name of the node are added to list B, and then the node in list A is deleted.

Because ZigBee belongs to wireless communication, there is instability. When the number of surrounding nodes remains unchanged, the result of the last search may be different from that of the next time. The interface will flash especially unstable in this way. This requires filtering. The first filtering scheme: search five times each time, and it is determined to exist if two or more times are found; otherwise, it is determined to not exist so that the nodes displayed on the interface are stable. But there is a problem; that is, the task has been searching, occupying ZigBee module resources; other tasks have to wait until the end of the search task to access which is not real-time enough. The second filtering scheme is to establish a queue, put the searched node at the end of the queue each time, delete the node information at the head, and move the queue members next time, that is, move the first one to the position of the second queue member and do it in turn. The number of member nodes of the queue is four and remains unchanged. As long as there are two or more members with this node, it will be judged that this node exists. In this way, each search can make it displayed on the interface, the number of searches is reduced, and the time of occupying the ZigBee module is four times less than the previous scheme. There are more opportunities for other tasks to not only occupy the ZigBee module but also improve the real-time performance of the task, but the disadvantage is that it takes about 3 seconds to remove a node. But this has little influence and the second design scheme is finally selected based on the practice.

In order to ensure the correctness of multitask execution, the function of real-time temperature and humidity detection is added. It only needs to ensure that the temperature and humidity verification is successful once in one minute. Otherwise, it takes 40 times to read the temperature and humidity data, and the reading of each bit of the data needs to delay to judge whether it is high level or low level. When this delay occurs, the CPU will switch to other tasks to improve its utilization, but when the delay time expires, due to the priority of the task, the task did not switch back on time which finally led to the unsuccessful verification of the measured data.

### 4.2. Debug of Light Control Node

#### 4.2.1. Dimming with PWM Wave

Pulse width modulation (PWM) is the time of high level output in a cycle. The adjustment of the duty cycle is the modulation of the pulse width. The color brightness of different RGB of duty cycles is different. Different colors can be modulated by outputting different PWM waves to the three channels of red green blue. The PWM module is like a timer built in. First, a count upper limit value (cycle) is set; then, a time *t* is set. When the counter is less than *t*, it controls the output low level of IO port. When the time is higher than *t*, the output of IO port is controlled as high level. When the counter reaches the upper limit, it will time again. So repeatedly, IO port outputs rectangular waveform [24].

#### 4.2.2. Light State Adjustment

The adjustment of color is mainly realized through the three primary colors of red, green, and blue. The RGB lamp can be configured with 256 different colors which depend on the adjustment of three PWM waves that control red, green, and blue. When the user sends the color switching command on the color selection panel of the control panel, the lamp control node will realize the color preparation by controlling three PWM waves of red, green, and blue.

Brightness adjustment: only change the brightness of the light, and keep the original color unchanged. The duty cycle of red, green, and blue PWM is equal to the increase or decrease of the column.

The flashing frequency of light can be controlled by a software program. The principle of flicker is to continuously control the light on and off showing certain regularity. The delay time between on and off is different with different flashing frequencies.

The gradient effect is realized by sending corresponding instructions from the controller. The lamp control node will make the lamp produce the effect of breathing lamp, slowly turn on, and then slowly turn off, and so on when the controller sends the gradient command.

Scene Effects: three different scene effect modes. Scene Effect 1: red, green, and blue lights turn on and off alternately, and with the passage of time, the speed of alternation is faster and faster. Scene Effect 2: use a random generating function to configure the color to be random and change once per second. Scene Effect 3: the breathing effect of red, green, and blue lights. The scene effect of each scene control signal is changed once.

Timing switch: the timing control lamp is on and off and is timed by the control end. When the preset time is over, the controller sends the control information of the timing switch.

### 4.3. Optimization of Light Control Node Real-Time State Switching

When debugging the light control node, the control panel keeps sending control commands, but the light control node does not switch state in real time. Instead, when one state is executed, it will receive another information and then execute it. When a sent state is executed for a long time (scene effect), if the control interface sends another state (color switching) during execution, but the light control interface does not execute the latest sent state (color switching), it will continue to wait until the last state (scene effect) is executed. This will make people feel as if the command sent by the user has not been executed. Because the ZigBee module does not support interrupt, it can only use software to improve the real-time performance of the execution state. When executing a task (Scene Effect 1, red green blue cycle switch), the red light is on, keep the current execution state, exit this state, and go back to see if there is a control command received; if not, continue to execute the previous state and then switch the light to green. At this time, if the new control information is received, the control will be executed. This greatly increases the real-time performance of task switching and can interrupt the current task state to execute another state.

## 5. Summary

This design mainly involves ZigBee wireless communication technology, task allocation, and management of the operating system, resource management, and communication between multiple tasks, emWin interface design, PWM wave control of RGB lamp status, serial port debugging, and j-link debugging skills. The system supports ZigBee protocol communication, supports the human-computer interface, controls the light on and off, and controls the light brightness and color, gradient, flashing frequency; supports timing switch, and scene mode. It supports the automatic addition and deletion of lamp control nodes, can display indoor temperature data in real time, can stably display node information, can freely modify the name of node display, and identify nodes with the same network address. The design has a wide range of applications. It provides technical support for the application of ZigBee technology in the field of light control.

## Data Availability

The data used to support the findings of this study are available from the corresponding author upon request.
